# The HBIM-GIS Main10ance Platform to Enhance the Maintenance and Conservation of Historical Built Heritage

**DOI:** 10.3390/s23198112

**Published:** 2023-09-27

**Authors:** Francesca Matrone, Elisabetta Colucci, Emmanuele Iacono, Gianvito Marino Ventura

**Affiliations:** 1 Department of Environmental, Land and Infrastructure Engineering (DIATI), Politecnico di Torino, Corso Duca degli Abruzzi 24, 10129 Torino, Italy; 2 Department of Architecture and Design (DAD), Viale Mattioli, 39, 10125 Torino, Italy; elisabetta.colucci@polito.it; 3 Department of Structural, Geotechnical and Building Engineering (DISEG), Politecnico di Torino, Corso Duca degli Abruzzi 24, 10129 Torino, Italy; emmanuele.iacono@polito.it; 4 Interuniversity Department of Regional and Urban Studies and Planning (DIST), Politecnico di Torino, Corso Duca degli Abruzzi 24, 10129 Torino, Italy; gianvito.ventura@polito.it

**Keywords:** spatial database, scan-to-BIM, HBIM-GIS, interoperability, CityGML, IFC, LOD, web platform, multiscale approach, maintenance, cultural heritage

## Abstract

This paper aims to describe the outcomes of the Main10ance project, which focused on developing an integrated HBIM-GIS platform to support the maintenance and conservation plans for the historic built heritage. The pilot case is the UNESCO complex of the *Sacri Monti*, located in northern Italy and Switzerland, which consists of groups of chapels and architectural artifacts holding significant historical and cultural value. Given their importance, specific maintenance plans involving multiple stakeholders and specialists are required. This study focuses on creating a unified system that semantically and spatially describes the architectural elements of the case study and the surrounding context and indoor assets. After a 3D integrated metric survey and the related data processing, parametric 3D models were created in a BIM environment, and a spatial database was developed to incorporate both geometric and alphanumeric entities. The database enables interoperability among different actors and domains, gathering heritage-related information crucial for restoration and conservation purposes. Additionally, the custom 4MAIN10ANCE web platform was developed with different levels of details (LODs), enabling the retrieval of both 2D and 3D data and establishing connections between the BIM models of the chapels and associated information.

## 1. Introduction

IT systems provide a variety of tools and solutions that can significantly improve the management, conservation, and preservation of architectural and cultural heritage. By embracing technology, heritage workers may better understand, document, conserve, and disseminate both historical and aesthetic significance. Therefore, on one side, GIS (Geographic Information Systems) technology may be utilized to produce digital maps that overlay historical landmarks and structures, aiding the comprehension of spatial relationships, planning conservation activities related to the environmental context where the cultural heritage is inserted, and analyzing potential risks. Conversely, the BIM (Building Information Modeling) domain may contribute to more detailed analyses of the architectural components and the indoor elements.

When preserving cultural heritage, one of the primary goals is to find solutions that minimize interventions in the long term while maintaining its function, value, and authenticity. *Planned maintenance* is an effective means to achieve this objective by carefully and meticulously planning interventions and activities, ensuring the legacy and continuity of the asset. This process should also incorporate rigorous and accurate timing to prevent the development of pathologies or risks that could impact the asset’s condition over time.

Based on these principles, the *Main10ance* project “https://main10ance.eu/” (accessed on 23 June 2023) aims to simplify the collection, archival, management and visualization of information pertaining to the asset and its conservation activities, both past and future. This endeavor was undertaken through the pilot case of the *Sacri Monti* system, a UNESCO World Heritage site since 2003. The Sacri Monti system serves as an exemplary instance of widespread heritage with inherent complexities, where the outdoor environment, architectural structures, and indoor assets contribute to its fathomless value. The development of such a new holistic *information system* enables its scalability to other similar contexts. By addressing the management challenges associated with this complicated and multi-scale heritage, which encompasses various domains, including artwork, buildings, and landscapes, the chosen case study exemplifies the design and implementation of a solution (an interoperable HBIM-GIS database connected to a web platform) useful for both professionals and the public management bodies. Furthermore, it allows for exploring tangled and tricky issues related to restoration and conservation activities, as well as the critical aspects of its management.

**Figure 1:**
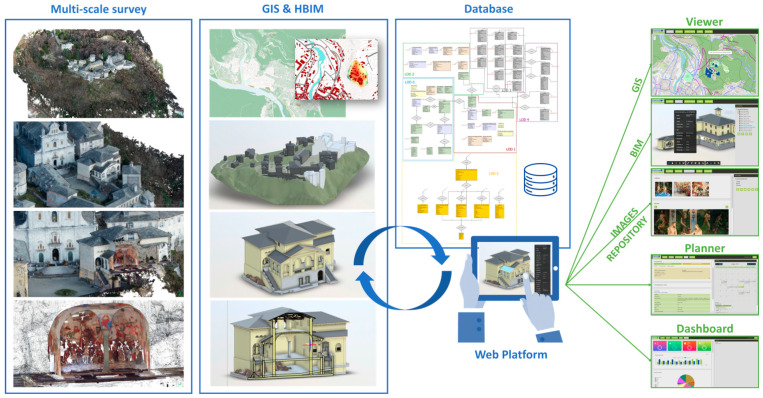
The Main10ance workflow from the 3D metric surveying activities to the final web platform realization, as illustrated in [3].

In this framework, the research presents the whole workflow (Figure 1) from the 3D metric surveying activities (Section 2.2.1), the scan-to-BIM process and the HBIM (Historic BIM) modeling phases (Section 2.3), the designing of the multi-scale database (Section 2.4) and its effective development (Section 3.1), to the final results, as the structuring of the web platform (Section 3.2) and its connection to the database.

In particular, this contribution details the preliminary steps described in [1,2] and the final web demonstrator [3]. With regard to the online platform, it allows viewing and interacting via an ad hoc interface, with all the data integrated within the DB. It is important to highlight that the *4MAIN10ANCE platform* is web-based and accessible via workstation or mobile. No specific software is needed, no license, and the source code is freely accessible. The source code is available at: https://github.com/EmmanueleIacono/MAIN10ANCE_Viewer_DEMO (accessed on 23 June 2023). The repository also contains a constantly up-to-date link to the actual web platform. It is also cloud-based, so all information is kept online with real-time updating.

### Related Works

One of the main objectives of the Main10ance project is the structuring of a solution interoperable between different representation scales, models, and platforms. In this regard, a state-of-the-art survey was conducted regarding the integration of GIS and HBIM domains and their adoption for planned maintenance activities.

The database is the core of the methodology, capable of connecting the territorial GIS representation with the building representation, HBIM, specific to churches, chapels, and movable assets of the various Sacri Monti complexes (Figure 1).

In the current scenario, BIM-GIS integration has become increasingly important, thanks to the possible geometric, technical, and semantic harmonization of the micro-scale of buildings (BIM) and the territorial-city level (GIS) representation. In recent decades, several studies, such as [4], emphasize the need to integrate these two domains for different purposes and analyses.

Their integration is not a new idea [5,6,7,8] and is the subject of international benchmarks such as GeoBIM [9], where conversion procedures between IFC and CityGML have been studied. The potential for smart cities, sustainable environments, or the construction sector are some of the many positive implications [10,11,12]. However, relying on a single database that conforms to standards, combining the informational levels of both environments is a relatively new approach.

In analyzing the applications of BIM and GIS, one of the first challenges to tackle is how to solve their technical integration.

CityGML (Geography Markup Language) is probably the most internationally recognized standard data model for representing multi-scale 3D information for buildings and cities. On the other hand, INSPIRE (Interoperable Spatial Data Infrastructure in Europe) is the European directive (published in 2007 and mandatory in all European countries since 2020) aimed at creating an interoperable territorial data infrastructure in Europe. Regarding architecture, engineering, construction, as well as resource and facility management, the Industry Foundation Classes is the standard designed for the Building Information Model (BIM). The IFC standard, certified by Building SMART, provides an object-based view of the model, including 3D and 2D geometries, along with the relationships between objects. It aims to represent parametric information. These standards have been considered as the basis for database structuring.

Moreover, in the domain of the CityGML, the Main10ance database followed the Levels of Detail structure (version 2.0). Hence, another aspect to focus on in analyzing BIM and GIS domains is LoD. In GIS, LOD refers to the level of detail [13,14], while in BIM, it mainly refers to Levels of Development (LoD). In CityGML, LODs are divided as follows: LOD 0 represents the regional and landscape level, LOD 1 represents the regional level, LOD 2 represents the district and urban context, LOD 3 represents the external architectural model and landmarks, and finally, LOD 4 represents the internal architectural model. However, LOD 4 (in version 2.0 considered during the project) was not sufficiently accurate to represent building information like BIM does.

For this reason, the architectural scale considered in the Main10ance database is a novel application and integration of these standards for the cultural heritage sector, including LOD 2-3-4 of CityGML and from LoD A to LoD F of IFC. In addition, a further new level of detail, LoD5 has been purposely created for this project.

Lastly, among the sources cited for the definition of the Main10ance database, there is the BDTRE (https://www.geoportale.piemonte.it/cms/bdtre/bdtre-2, accessed on 16 September 2022), the Reference Territorial Database of the Piedmont entities. The vector data were downloaded from BDTRE conform to the INSPIRE standard and were collected in the GeoTopographic Database (2019 vector dataset) at a scale of 1:10,000.

Concerning the various outputs of the Main10ance project, to the best of our knowledge, no existing platforms are currently available which combine the potentialities of an integrated HBIM-GIS interoperable database (structured according to specific restorers and management bodies’ necessities for the planned maintenance), connected in real-time to a freely available web platform, with the direct exploitation of HBIM models and GIS data directly obtained by a 3D metric and integrated survey. Nonetheless, a step in this direction has been made in the works of [15], where a vulnerability-oriented GIS/HBIM integration in an urban 3D geodatabase is proposed, ref. [16] with a database that integrates HBIM and GIS, including information about historical data and the status quo of infrastructure heritage [17], who suggest a procedure for the generation of 3D visualization models of existing cities by integrating HBIM models in GIS environments, ref. [18] that developed a web information system able to integrate BIM and GIS data, with particular focus on the analysis of the historicized city and its main buildings over time.

Therefore, this research is unique because, from the beginning of the Main10ance project, many experts, such as technicians, restorers, the management body, and administrations, have been involved in discussions and preliminary studies about the real needs and operations for the maintenance and restoration activities of the case study. This element constitutes a great strength of the proposed work. Then, the whole database, with its entities, has been designed following these suggestions, from the level of detail 0 to level of detail 5. Parallelly, eight standards have been adopted, making the research reusable and interoperable.

Moreover, this research is distinctive and innovative because, for the first time, a digital platform integrating BIM and GIS, with a common database structure behind it, has been developed starting from an accurate integrated 3D metric survey. The platform and the related DB could be easily queried and updated by technical operators and non-experts, and it could be widely adopted for other cultural heritage case studies.

As previously mentioned, the present paper does not particularly focus on the database structure, already described in [2,19], but it aims to underline the overall workflow followed to develop the web application, used as a demonstrator, in which the BIM and GIS domains have been integrated and connected with the database. According to the five LODs (Levels of Detail) specified by the standard, an additional LOD 5 has been added specifically for this project. This LOD encompasses all tabular data (non-geometric entities) related to maintenance on the *Sacri Monti*.

## 2. Materials and Methods

### 2.1. The Case Study

**Figure 2:**
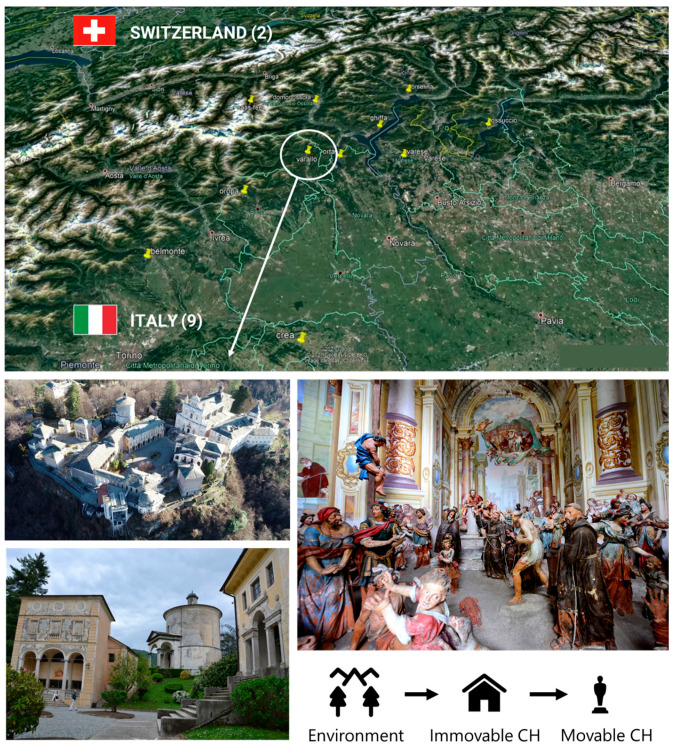
The eleven Sacri Monti (**top**) and a focus of the Sacro Monte of Varallo Sesia with the mingling of the environmental context, immovable and movable cultural heritage.

One of the main objectives of the project is to focus on the maintenance plans of the *Sacri Monti* of northern Italy and Switzerland. These complexes are groups of chapels and other architectural artifacts, such as statues, frescoes, wall paintings, and terracotta sculptures. They were primarily built between the 16th and 17th centuries. These architectural assets hold significant value and are situated in mountain landscapes and environmental settings. In 2003, they were recognized as part of the UNESCO World Heritage List in Piedmont and Lombardy, comprising 7 sites in Piedmont, 2 sites in Lombardy, and 2 sites in Switzerland (Figure 2). Each chapel contains statues, frescoes, and iconographic elements, which contribute to the complexity of overall management and maintenance.

They have been chosen as case studies of the Main10ance project since the management body strongly wanted a system able to properly manage the diverse data available and able to support and facilitate the Planned Maintenance Plans.

The Interreg project “Main10ance” thus aims to develop a working method and operational tools to guide clients, management and control entities, and professionals in developing a planned conservation plan for cultural heritage based on the principles of sustainability, with a long-term vision of the planned interventions and optimizing the available resources. The project was co-financed by the Interreg V-A Italy-Switzerland Cooperation Program through the European Region.

### 2.2. The 3D Integrated Metric Survey

The methodology described below was applied to three Sacri Monti, in particular those of Orta, Ghiffa, and Varallo. By way of example, some activities of the surveying campaigns carried out at the Sacro Monte of Varallo are outlined (for a detailed comprehension refer to [20,21]. However, the results obtained and the process followed were the same for the various sites.

The particular arrangement of the 45 chapels of the Sacro Monte of Varallo Sesia on a rocky outcrop made it necessary to use a multi-sensor approach with both image-based and range-based techniques. The GPS/GNSS positioning system and the total station have allowed the definition of the framing and detailed network, with precision topographic measurements. The UAVs (Unmanned Aerial Vehicles) were used for the acquisition of aerial photogrammetric data on areas otherwise difficult to reach, and, finally, the Time-of-Flight terrestrial laser scanner systems were useful for the more accessible parts of the site and for the indoor environments of the chapels. Furthermore, in this context, the use of MMS (Mobile Mapping Systems) and low-cost sensors, and COTS (Commercial Off The Shelf) have also been tested, allowing the reduction of acquisition times and speeding up survey operations [22].

The results of these surveying activities and the subsequent data processing phases were the creation of the point clouds of the chapels (which have been used as the base for the scan-to-BIM process), orthophotos, and digital elevation models.

The HBIM products thus created have been enriched with various types of information such as: technical-constructive aspects (materials, forms of alteration and degradation), historical reports and documents, etc. Finally, this information was exported to allow for the structuring of an external relational database.

#### 2.2.1. Data Acquisition and Processing

The data acquisition activities took place in several survey campaigns from 2015 and 2021. As previously mentioned, during the survey campaigns, a part of the framing network vertexes was surveyed with the GPS/GNSS technique in static mode, while some refinement points, undetectable due to poor satellite coverage or the impervious conformation of the places, were measured with the classic topographical technique. Once the data on the framing and refinement network had been acquired, it was possible to proceed with the detail network. To obtain dense metric data, laser scans were performed both indoors (attic, porticoed areas, loggias, and chapels) and outdoors. In total, more than 100 scans were performed with a resolution of 1 point every 5 mm.

However, since some areas were not fully accessible due to the orography and the prominent position of the complex, it was necessary to resort to the use of UAV to survey not only all the roofing systems but also all those parts of buildings otherwise unreachable. For this purpose, several nadiral (90°) and oblique (45°) flights were planned for an intake area >38,000 m^2^.

**Figure 3:**
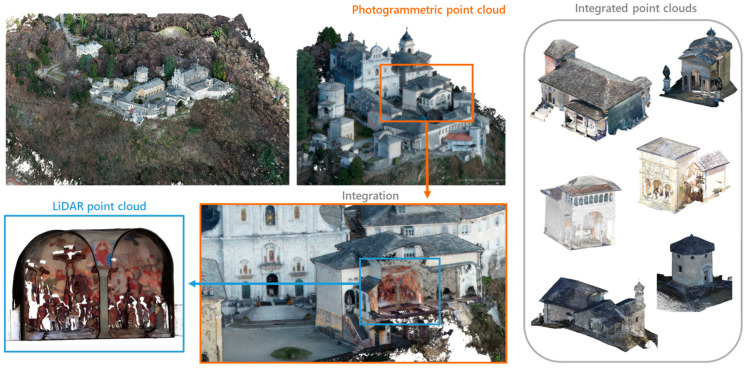
Integration of the photogrammetric point cloud with the LiDAR ones to obtain complete data.

Finally, MMS Stencil Kaarta (Figure 3, bottom right) was used to acquire the ground data under the tree crowns. This made it possible to integrate the point cloud acquired by drone, where there was a strong lack of data, for the creation of the DTM.

Following the acquisition phases, the photogrammetric data and laser scans were processed, ensuring residues on the markers were less than 10 mm. This threshold was set on the basis of the desired final representation scale of 1:100.

As regards the photogrammetric data, different types of software were tested and the resulting point clouds were compared (C2C—Cloud to Cloud, and density) to establish which was the best solution. More specifically, over the years, ContextCapture by Bentley System, Agisoft Metashape, Visual SFM, Pix4D, and MicMac have been used.

In any case, after processing the point clouds, comparing, cleaning, and filtering them, the data fusion process was carried out, which made it possible to integrate the LiDAR point clouds with the missing parts (roofs or inaccessible areas) deriving from the photogrammetric ones (Figure 3).

In addition to the point cloud processing, the DTM (Digital Terrain Model) was also created. This model constituted the topographic surface in the object-oriented software.

**Figure 4:**
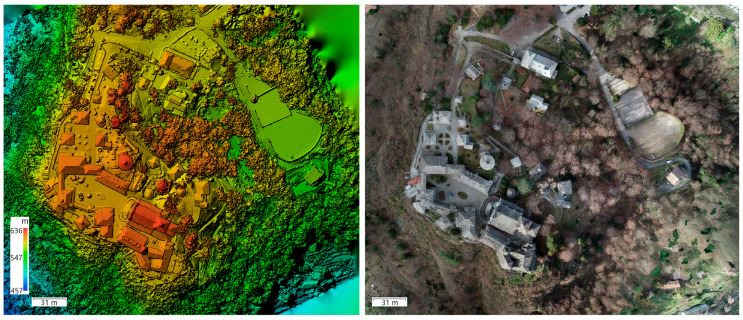
DSM (**left**) and orthophoto (**right**).

Finally, from the photogrammetric point cloud, an orthophoto of the complex with 4 cm pixels and the DSM (Digital Surface Model) were also created (Figure 4).

**Figure 5:**
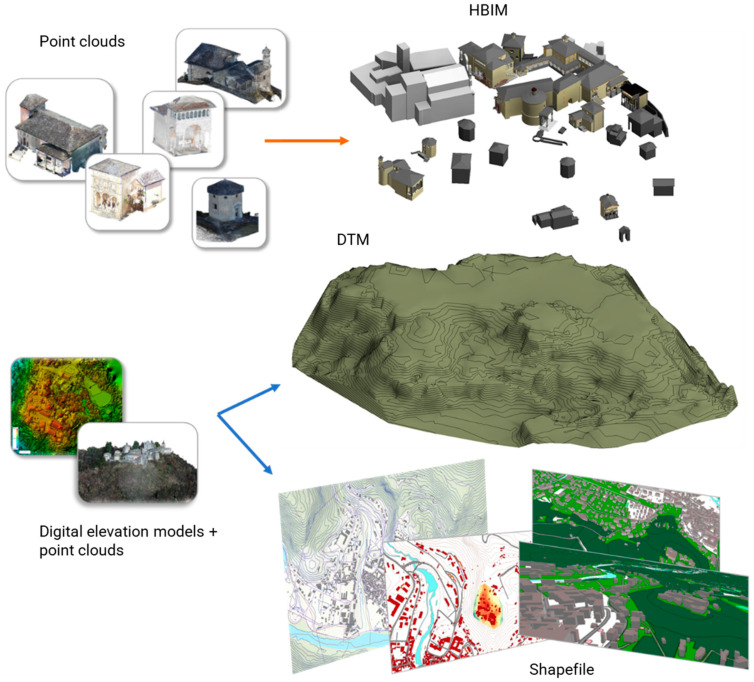
Data workflow from point clouds to HBIM models and GIS environment.

The set of these data was then integrated into the Main10ance database (through GIS data and HBIM models) (Figure 5) and established a basis for the survey of the current state, which can always and easily be updated in the future.

### 2.3. The Scan-to-HBIM Process

#### 2.3.1. Modeling Principles

The scan-to-BIM process was one of the key elements of the methodology described here, as the maintenance platform studied and developed (Section 3) heavily relies on the use of three-dimensional models structured using the BIM methodology. This happens since each element belonging to a specific building, properly modeled, corresponds to certain parameters related to specific characteristics of the building.

The first step of the process was to adopt the most suitable formats for BIM modeling. Having chosen to carry out the latter using Autodesk Revit software (version 2020), it was necessary to convert the point clouds processed following the survey campaigns, received in .E57 format, into one compatible with such software. Autodesk Recap was used for this purpose, through which point clouds can be manipulated, divided, and cleaned, and then imported into the modeling software as a .rcp files. These point clouds are also already correctly spatially geolocated, which means that the models produced from them will be as well. Regarding the modeling process of each building, during the earlier stages of the project, an additional step consisting in the segmentation of the point clouds into individual architectural elements was initially experimented with in order to facilitate the actual modeling steps. However, this step was later skipped for other cases, and the point cloud was imported within the BIM authoring software in its entirety since modeling with the aid of section boxes and similar tools was deemed to be more flexible. Considering the two strategies equal in terms of results, the latter was opted for, being the most time-efficient.

Modeling via Revit was carried out following two different levels of detail: The first modeling for the general volumes of the buildings (identified internally to the project as “LOD2”) was performed by modeling each Sacro Monte in a single model, in which the context ground, the general volume of each building and the roof, modeled separately, are present. The next level of modeling concerned the creation of models for each building belonging to a certain Sacro Monte at a higher level of detail (so-called “LOD3”). The established level of detail was fundamentally a compromise between the two different purposes of the model uses: The modeling principle was thus, on the one hand, the use for conservation, and on the other hand, that of visualization for tourist and promotional valorization purposes. In this case, starting from previously agreed standards and guidelines within the research project was essential, relating to the subdivision of artifacts into distinct and properly coded building components.

In particular, since the models produced in the context of the research project were intended for use in maintenance management, it was deemed appropriate to establish specific categories of elements through which to organize the multiple categories of objects native to the modeling software. For example, there is no “Vault” category in Autodesk Revit software. Moreover, this element can be modeled through different system families such as “Wall” or “Floor”. Since vaults are a very common architectural element of historic buildings, and are also quite important for preservation purposes, therefore deserving of their own entity within the database, it was decided to identify these elements, and others with similar issues, through an ad hoc identification code, which is described in depth in the next section. This was useful both for simplifying their management within the unified maintenance database and ensuring the highest possible correspondence with certain “risk expression”, which were prepared ad hoc by colleagues involved in the restoration and maintenance disciplines of the research project.

Such risk expression essentially represents a classification of the possible risk elements to which artifacts, parts of them, or their surrounding context, may be subject. From them, a series of corresponding periodic control and intervention actions to be carried out on the artifacts mentioned above, as well as any corrective actions in case of critical issues, damages or ongoing degradation of the elements, can be rigorously derived [23,24].

#### 2.3.2. Standardizing the Classification of Asset Elements

Once the categorization of the above elements was determined, the next step was to define a unique identification code for each individual element, such that even from a simple reading of the code of an element, its belonging could be understood. For this reason, it was decided to adopt a logical breakdown of the main identifying data of the elements based on a hierarchical breakdown into *four levels*: site or context of belonging, building, maintenance category, and numerical identifier.

As for the *first* of these levels, it was decided to adopt a three-to-four-character code that would allow for relatively easy identification of the reference location. Therefore, in this case, for example, all objects located at the Sacro Monte of Varallo are characterized by the code “SMV”, just as those belonging to the Sacro Monte of Ghiffa have the code “SMG”, etc., for all the other locations under study.

The *second* level of the identification code is that of the “container” building of the elements. In the case of the Sacri Monti, it was considered that the easiest way to identify the various building artifacts, also for the purposes of the application that will be discussed later, was to indicate the number of the chapel contained within the respective building.

Since the detailed BIM modeling was carried out for buildings, this identification code component proved fundamental to tracing the belonging BIM model of the aforementioned components easily. Furthermore, since sometimes the same building contains more than one chapel, not necessarily consecutive with respect to the devotional path, it was established to indicate the entire numerical sequence of chapels to avoid misunderstandings. Thus, for example, the building of the Sacro Monte of Varallo hosting chapels from no. 5 to no. 9 would have the code “5–6–7–8–9”, while the one hosting chapels no. 16 and no. 24 would have the code “16–24”. It makes the content of the building artifact extremely clear and facilitates the operations of ID breakdown and searches within the shared database based on this breakdown. The only exception to this rule was the group of non-modeled elements belonging to the so-called LOD4, i.e., elements belonging to specific chapels, such as statues, paintings, etc., and, as for the LOD3, i.e., modeled objects, those belonging to the category of “grates” as an interface of specific chapels and thus elements of “passage” between one LOD and another. It is evident that for different case studies, it is naturally appropriate to establish further meaningful codes or terminology.

The *third* level of the hierarchical breakdown concerns the category of the element, according to the logical breakdown previously established for risk expressions and related operational actions. This is the ID level through which it is possible, starting from the planning of conservative operations, to identify and select the individual elements concerned by such operations and to associate them with certain intervention sheets, which will be discussed later.

Finally, the *fourth* and last level, the most specific one, is essentially represented by the numerical identifier of objects (normally consisting of five or six digits depending on the object) automatically assigned by the modeling software, which guarantees the uniqueness of each individual ID within the database. The identification number used alone, not in combination with the previously described levels, did not provide the same guarantee of uniqueness due to the fact that in two different models composed of hundreds, sometimes thousands, of elements, there was often the possibility of repetition, resulting in overlap within the shared database.

#### 2.3.3. Automating the ID Compilation

The identifier, “id_main10ance” [25,26], is created as a parameter associated with all the elements modeled using the BIM methodology and set as an attribute in all the entities of the shared database representing the previously established maintenance categories. It is structured as a single text string composed of each of the four hierarchical levels described, connected by a separator character (in this case, the character “|”, Unicode: U + 007C).

In the case of BIM models, which often consist of several hundred elements, it is not possible to manually insert such an identifier for each element, and even if it were possible, errors or inaccuracies caused by the repetitiveness of manual input are highly probable. The Dynamo plugin present within the Autodesk Revit software was used to solve this critical issue. This plugin allows the combination of so-called nodes, each of which performs a pre-set elementary operation, to compose scripts capable of performing previously unavailable actions with the standard modeling tools [27]. Thanks to these additional functionalities, manual input was reduced to the massive compilation for selected groups of elements of the correct corresponding maintenance category. Moreover, the first two levels of the ID, i.e., the abbreviation of the location of belonging and that of the building, are always unique for all the elements belonging to a specific LOD3 model (thanks to the adopted modeling strategy explained above) and can be entered only once and repeated for each individual element. The script developed for ID compilation receives these common abbreviations as input from the user and then proceeds to read, object by object, its maintenance category (previously entered through selection groups in a dedicated text parameter) and its numeric identifier, automatically set by the modeling software.

Once all four code levels are collected, the script compiles the overall string, complete with separator characters between the different hierarchical levels, and inserts it into a text parameter called “id_main10ance” associated with all the model elements. At the end of the Dynamo script execution, all modeled objects are characterized by a unique ID that allows all the necessary maintenance operations within the platform to be performed and the corresponding elements to be correctly associated with them.

An important note concerns the choice to use a structured and ad hoc unique identifier instead of one of the many existing and standardized building component identification codes at both national and international levels. Specifically, the Italian UNI standard has a specific standard, UNI 8290:1981 [28], which classifies building components into three distinct levels. However, within the scope of the research project, it was evaluated that the logical breakdown provided by the standard was not sufficient to fully meet the project’s objectives, as the standard proposes a classification compatible with contemporary construction, whereas specific categories of additional elements are necessary for historical artifacts. Furthermore, the proposed code is characterized solely by sequences of numerical digits, which are difficult to understand with a quick reading by an interested operator, perhaps in the field. The above reasons also apply to the decision not to use international classification systems.

Finally, another aspect not to be overlooked concerning the association of maintenance IDs with BIM model elements is that there is a possibility that not all objects are coded but that some of them do not have the maintenance ID. This situation may be due to a failure to compile the category parameter, hence an error, or a conscious decision, justifiable because, since the models can also be used for first-person virtual exploration of buildings, there is a possibility that some elements may be modeled but not be relevant for maintenance operations. However, it is essential to pay adequate attention, for example, through appropriate checks using view filters, group selections, or other methods, to ensure that all elements to be included in the shared database and maintenance cycle have not only the ID completed in all its parts but also that these are methodologically correct. In fact, once the model is loaded onto the platform developed for maintenance, the planned conservation cycle will never be started for non-coded elements, and modifying this condition by directly acting on the internal model parameters is not an action possible from the platform itself. Instead, it is necessary to remove the model from one’s cloud storage and reload it, which is not a trivial task.

### 2.4. The Multi-Scale Database Design

**Figure 6:**
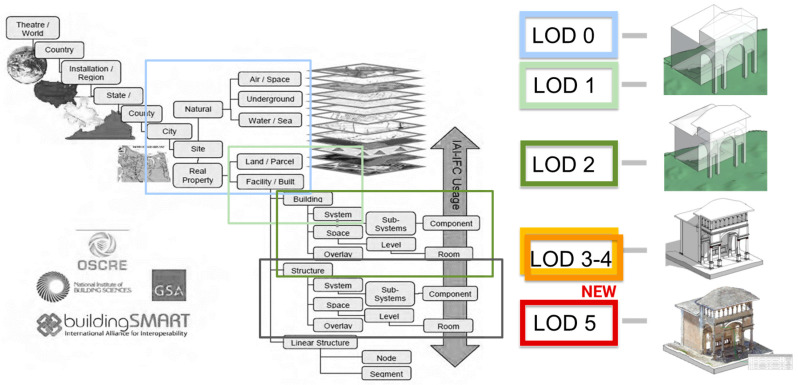
The LoDs of the Main10ance database in relation to the information represented according to the *BIM buildingSmart*.

Before implementing the DB based on the aforementioned levels of detail, the preliminary phase of database modeling aims at selecting an appropriate data model to manage the data on the specific application domain. Various information from multiple actors, stakeholders, and technicians has been collected to accurately represent the domain of historical buildings and structure the DB according to user needs. The subsequent modeling phases have followed the standard workflow with the definition of an external model, a conceptual model (from LoD 0 to LoD 4—Levels of Detail—as depicted in Figure 6), a logical model, and an internal model (the database). The classes selected for the DB definition are subdivided according to Ci-tyGML LoDs. These classes partly derive from BDTRE and from various analyses and discussions among project partners, aimed at fully defining the objects of the Sacri Monti that require maintenance actions (scheduled or ordinary).

**Figure 7:**
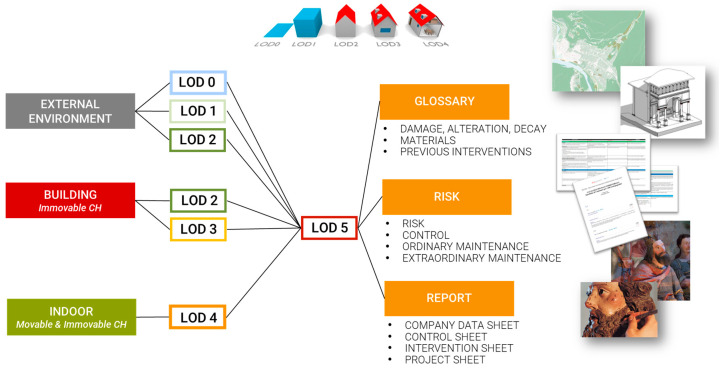
The different LoD according to the CityGML definition and the corresponding content in the Main10ance database.

LoDs 0–2 provide rather contextual data and represent an urban scale. LoDs 2–3 define the external environment and architectural components, LoD 4 represents internal elements, and LoD 5 contains alphanumeric data about maintenance plans and conservation status (Figure 7). Depending on its definition, each geometric entity has been classified as belonging to a lower or higher level of detail (LoD), following the CityGML standard. Considering the project’s purpose of creating a management model for the maintenance of Sacri Monti, LoDs have been linked to the macro-categories of External Environment, Buildings, Real Estate, and Furniture, which are subsequently connected to the new LoD 5 containing alphanumeric data related to maintenance activities for buildings, chapels, etc.

## 3. Results

### 3.1. The Multi-Scale Database

**Figure 8:**
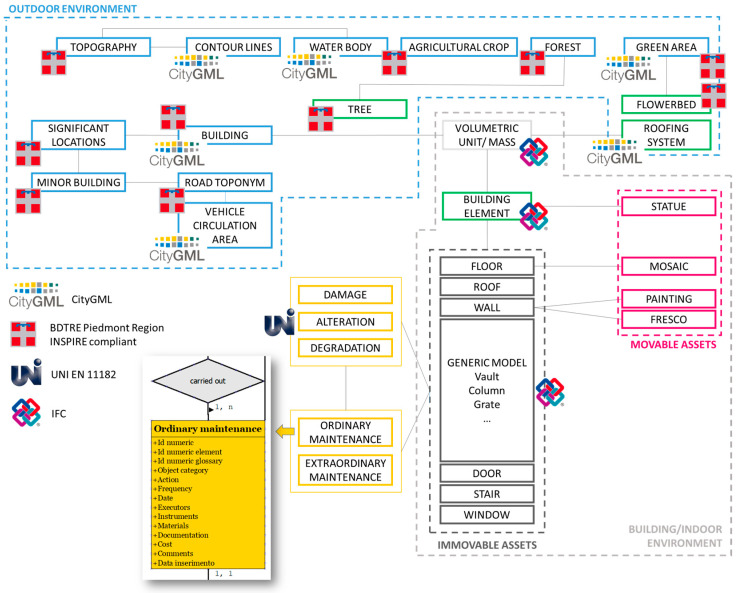
Conceptual schema of the DB and example of the “ordinary maintenance” table in the Conceptual model. The dotted lines represent the main clusters of the LODs.

The real world and objects of the Sacri Monti have been conceptualized and transformed into models by selecting various entities from the standards and the BDTRE. In the conceptual model, the entities to be managed in the database, their attributes, and their associations are formalized. The research considered the data models INSPIRE and OGC CityGML for this step. LoDs were adopted to design the conceptual model for creating spatial databases. The *IfcBuildingElement* connects urban data to architectural data through the mass/volume unit, i.e., CityGML with IFC. Most GIS entities refer to the BDTRE, which is INSPIRE-compliant. In contrast, data related to the conservation status refer to UNI 11182:2006 and a specific glossary developed by the partners, considering different Italian, Swiss, and international terminologies. Below, there is a conceptual schema of the database and the different sources of the entities (Figure 8).

Each LoD is intended for a different audience. LoD 0 to 5 are used to store information for the management of the Sacri Monti by park managers and public administration. LoD 0 to 2 are for tourism and green maintenance personnel. Finally, LoD 3 to 5 are used to store and update maintenance information by restorers, artisans, and professionals. The conceptual model also includes all the attributes of the entities. Some fields have an “enumeration,” which is a dropdown menu that allows you to select and fill in the field of the database according to a defined list.

Each entity created ad-hoc for the project, is designed to host various spatial and non-spatial objects representing the *Sacri Monti* and their context. These objects have been divided into LoD based on the level of representation and information. LoD 0 accommodates 2D data, such as DTM, contour lines, hydrography, green areas and forests, transportation, and infrastructure. LoD 1 has been populated with orthophotos, trees, volumetric units, and secondary roads. LoD 2 represents the 3D component at the territorial and city level, encompassing the coverings, flowerbeds, and volumetric masses of individual chapels. LoD 3 is intended for architectural elements (such as floors, roofs, walls, stairs, pillars, columns, etc.). Decorative elements are represented in LoD 4 through points, volumes, images, and external 3D models. Finally, LoD 5 has been implemented for fields related to risk, ordinary and extraordinary maintenance, and control.

Subsequent to the creation of the conceptual model, the logical model was developed to define the attribute types of the entities. These attributes were integrated after careful analysis and evaluations with various experts and can be:

- Integer numbers
- Floating-point numbers
- Character strings
- Sets of values to choose from (Enumeration)

Some fields derive from the abovementioned BDTRE specification and have been repeated for multiple entities, such as acquisition date, update date, data owner, supplying entity, and acquisition scale. Specific attributes have been integrated after careful analysis and evaluations with various experts. Examples of specific fields include forest types, tree species, place names, road types, pavement, and others.

Following the standards and data model analysis, the spatial database was designed using the free and open-source relational database management system PostgreSQL (version 13) with the graphical interface PgAdmin 4 and the spatial extension PostGIS for GIS connection. The structuring of the database also analyzed different levels of access and users. In particular, the involved users need to analyze data at different levels: Tourists only need to query general data and information. The managing entity needs to examine all specific entities and practices, while professionals and artisans only need to access relevant parts and details. This multilevel structure is reflected in the database, organized according to the users’ needs and specificities.

Finally, the classes were populated with spatial and alphanumeric data derived from the BDTRE (such as buildings, building elements, roads, and vegetation from the regional geoportal). Additionally, data from integrated metric survey processing (Section 2.2), such as rasters, such as DSM or DTM and orthophotos, were added using the “raster2psql” command in PostgreSQL.

**Figure 9:**
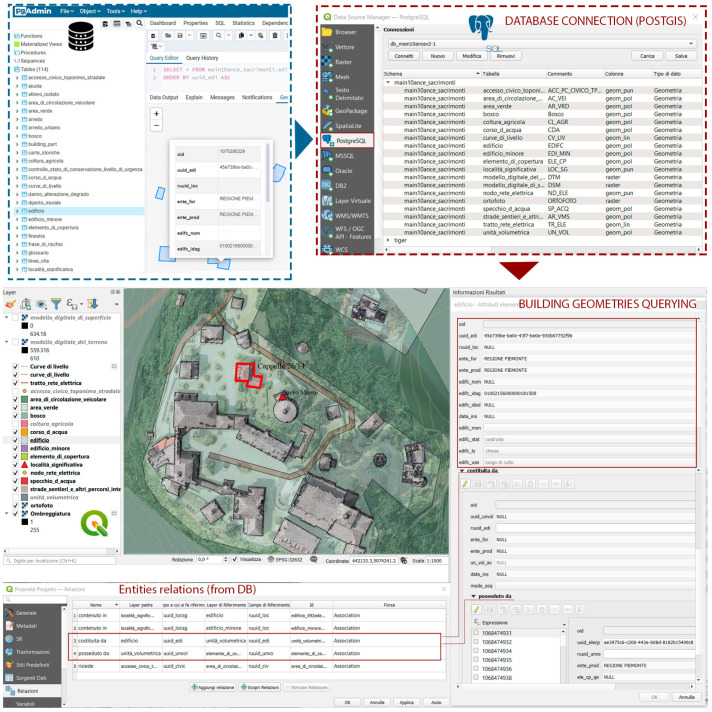
Database connection in GIS and geometries querying [2].

Finally, the database has been connected to the GIS through the spatial extension *PostGIS*. The software used is QGIS (version 3.20). In the GIS environment, it is also possible to import alphanumeric tables containing maintenance and risk-related information. The geometries can be queried, and thanks to the relationships implemented in the database, it is possible to access information from LoD 5 (Figure 9).

### 3.2. The 4MAIN10ANCE Platform

**Figure 10:**
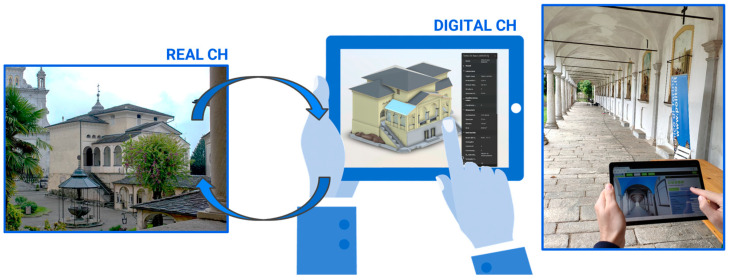
Realization of the workflow from the real to the digital heritage, available directly in situ through the 4MAIN10ANCE platform.

The following paragraphs describe the design choices made for the definition and structuring of the platform (Figure 10).

In particular, the preliminary steps performed to outline the general framework and requirements of the platform are illustrated in Section 3.2.1, Section 3.2.2, Section 3.2.3 and Section 3.2.4, while its effective implementation is detailed in the following paragraphs, from Section 3.2.5, Section 3.2.6, Section 3.2.7, Section 3.2.8, Section 3.2.9, Section 3.2.10 and Section 3.2.11.

#### 3.2.1. Data Domains

The design of the software architecture of the 4MAIN10ANCE platform developed for the project started from a series of considerations about which actions could be useful to perform on it, as well as on what should be a correct logistical organization of the actions on the artifacts. Firstly, enabling the visualization of all different types of elements present within the shared database. These are essentially divided between elements characterized by GIS-type information, meaning two-dimensional geometric elements (points, lines, polygons) with a series of associated attributes, three-dimensional objects modeled using BIM methodology (the so-called LoD3), also enriched with a series of characterizing parameters, finally, detail elements, typically contained within buildings and chapels, not modeled but important from the point of view of the maintenance plan (the so-called LoD4), represented by significant images and photos. This subdivision led to the intention to create three different *viewers* (Section 3.2.9) within the platform: a two-dimensional one with a map on which it could be possible to activate or deactivate the display of GIS elements, a second viewer, three-dimensional, within which to open, query and explore the three-dimensional BIM models of buildings, finally, a viewer for the last level, substantially characterized by a gallery of images that could be filtered by location, building, and category, and information sheets associated with each of these images.

#### 3.2.2. Structuring the Planned Conservation Data and Activities

**Figure 11:**
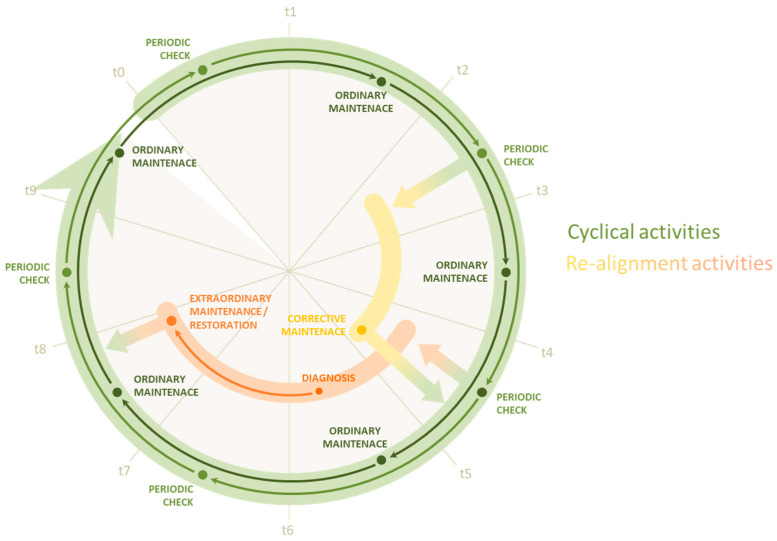
Maintenance cycle defined for the setting up of the activities to be inserted in the platform.

In addition to the possibility of displaying each type of element in the most appropriate way, it was also necessary to establish the most correct method of managing conservative operations on assets. For this reason, an attempt was made to imagine a *maintenance cycle* (Figure 11) that, starting from a state of relative operational and maintenance regularity of the elements involved, could somehow move away from this state and proceed along parallel paths if necessary, according to predetermined modalities based on what is required by Italian building regulations regarding possible interventions on artifacts [29].

In particular, it was imagined to start from a first level of basic operations, characterized by a periodic check of the elements, based on a relative period expressed in months, and according to what is provided by the previously introduced “risk expressions”, and by a possible, where provided, ordinary maintenance, a moderate-impact action on the asset. Following each check, the assigned technician verifies the correct correspondence of the asset (or parts thereof) to specific operational requirements, on the basis of which to record the state of its conservation. Based on these data, the maintenance cycle can continue with regular periodic operations or enter one of the “corrective” cycles provided.

These corrective cycles were essentially conceived as a temporary exit of the asset subject to intervention from the periodic control and maintenance cycle: During this time interval, the asset is subject to the most appropriate type of intervention with respect to its conservation status, according to what is provided by the regulations in terms of extraordinary maintenance and restoration interventions, and by the same risk expression in terms of guiding towards the most appropriate corrective intervention in case of relatively low criticality. Once the asset has had its good-conservation status restored at the end of these realignment activities, it is allowed to re-enter the normal cycle of periodic checks and ordinary maintenance.

The orientation towards the most appropriate corrective intervention for the asset in poor conservation status naturally derives from the level of poor conservation to which it is subject. It follows naturally that a more severe conservation situation must necessarily entail a more important restoration intervention in terms of cost, priority, and impact on the asset itself. This reading of the state of assets was the basis for the logic developed to design the algorithm that would automatically establish the most appropriate type of intervention with respect to the object’s level of conservation, according to what is reported by the operator.

Once digitized and entered into the platform, the property subject to scheduled maintenance can be in two possible situations: In an ideal T_0_, where all elements are in good preservation condition. In this case, by planning only cyclic control and maintenance activities, it will be possible to correctly initiate its maintenance cycle, subject to specific frequencies and regularly scheduled over time. Alternatively, the property may not be in good condition at the time its model is associated with the maintenance platform. Nonetheless, even for assets for which the situation is more burdensome or heterogeneous, it will be possible to plan an initial inspection, following which, the responsible operator will enter the necessary information about the element so that the most appropriate corrective maintenance or restoration activities can be derived from it, and which the system has identified as the most suitable realignment activities to restore its preservation condition.

#### 3.2.3. Applicability and Advantages of the Platform

Another fundamental concept in the design of the maintenance platform is the ability to access it at any time and from any place, not only from an office workstation but also possibly in the field, from a mobile device provided to technicians and operators, through Internet access to which the platform must necessarily be connected. It also follows that while each device can access it independently and allow users to perform different operations autonomously through the provided interface, the supporting database must be unique and accessible, in its different parts, by each device according to different needs. This common component of the platform must, therefore, be hosted on an appropriate server infrastructure that guarantees this level of accessibility.

All of this determines a series of clear advantages compared to the adoption of software used locally: In addition to the possibility, as mentioned earlier, of accessing the same data from different places and devices, this architecture also guarantees real-time data updates, facilitating collaboration between operators and avoiding the risks arising from manual management of information exchange and unnecessary duplication of data. Moreover, the development of an ad hoc system allows orienting the options of interactions made available with respect to the specific needs of each user, depending on the use that they must make of the platform.

The basis of each design evaluation that led to defining a functional structure for the platform, as described so far, lies in a set of specific operational requirements. They constituted a comprehensive set of needs that allowed the shaping of a concept that came as close as possible to the real use of such systems. Only after defining such a concept was it possible to proceed, with a clearer idea in mind, to the implementation of the most appropriate operational solutions relative to the required needs.

#### 3.2.4. Levels of User Authorization

A fundamental component of the set of operational needs identified for the conception of the platform is related to the definition of the different types of users it can potentially host and the possible interactions with its elements. Indeed, it emerged clearly from the beginning that, although the platform should be open to use by all potential interested parties, it is also necessary to establish specific rules and limitations deriving from the many actions they may be able to perform on it. Since there are different types of users, each with different roles and tasks, the application interface must be able to change depending on the user who interacts with it and provide the specific tools determined by their role. In particular, three categories of users were provided for, plus a fourth one above the other roles, with increasing permissions: tourist, operator, manager, and administrator.

The first level, that of the so-called “tourist user”, is that of anyone interested only in the tourist aspects of the assets: They are not a registered user on the platform and therefore do not need to perform any dedicated access, but they only use the basic visualization tools freely available to everyone. The possible applications can be various, such as promoting tourism in a specific location through the exploration of maps and model navigation. In addition, the three-dimensional aspect intrinsic to the platform provides the possibility of exploring models that can be both preliminary to an on-site visit, integrative with a visit already made, or alternative in cases where the interested user has specific difficulties, fragilities, or other limitations of motion.

The second type of user is the so-called “operator”, a figure that encompasses all professionals involved in asset management, from the maintainer to the restorer. This figure is allowed not only to view GIS elements, BIM models, and the image gallery but also to modify the related data: The operator can, in fact, enter new information, recording data related to inspection, diagnosis, intervention, etc., as well as modifying existing ones. For these reasons, this is also the first of the authorization levels provided, for which a specific registration on the platform and related login with their access credentials is required. The operator also has a specific area of interest, namely, it can only view and modify data relating to the asset or set of assets for which it has received a specific assignment. This is a measure that has been provided to ensure a certain level of data privacy about assets, as well as to ensure correct attribution of roles and responsibilities.

Finally, the “manager’s role” is that of those who administer, manage, and control all data relating to their management area. This level of authorization has permission for access to additional tools that allow them to intervene not only in data modification but also in the modification of the entire preservation process, modifying certain parameters related to the optimization of their maintenance activities. In addition, the manager must be able to access tools for supervising all data related to all assets under their responsibility.

A fourth figure, detached from individual maintenance processes, is that of the so-called “administrator”: this is a figure above the parties, impartial with respect to individual assets or various areas of application, possesses the highest level of authorization and deals with the management of the platform itself, taking care of its development and updating. It is again a question of maintenance, but in this case, not of the material component, but of the software component, which is also just as important.

#### 3.2.5. The Data Flow and Structure of the Platform

**Figure 12:**
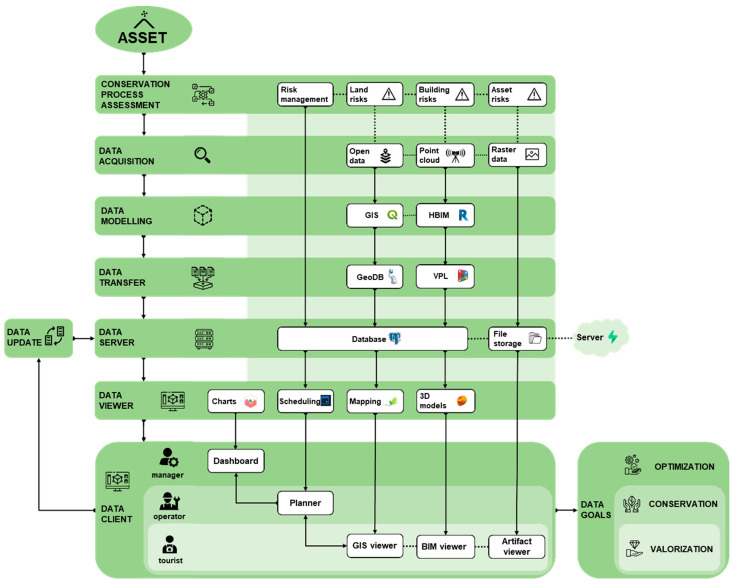
Conceptual representation of the data workflow and structure of the platform.

Once a general framework of requirements and a corresponding concept of how this should configure specific functionalities was established, the actual development of the maintenance platform could begin (Figure 12).

Firstly, it was decided to opt for the development of a web application instead of software to be run on a specific machine, both for the relative speed with which it was possible to create a prototype of the system and for the greater ease of implementing the necessary real-time connections with the shared database, which, in any case, would have to be hosted on an online server to communicate with the application being developed. The basic structure of it is divided into two main macro components: the *client* (also called front-end) and the *server* (also called backend). The former is made up of the interface, i.e., the pages, graphics elements, and everything with which the user interacts, through the use of a browser on which the system assembles such elements within a web page.

The second macro-element of the system is the server, which is the unique component that communicates with clients accessing the platform and handles requests and responses with them through HTTP protocol. The server also hosts the database, communicates with it, and extracts information from it to provide to the client. For the programming of the application, Microsoft Visual Studio Code was chosen as a text editor for its flexibility and ease of use, but this choice is in no way binding for the development of similar systems.

#### 3.2.6. The Web Platform Server

From the point of view of server implementation, *Node.js* was first chosen as the runtime environment: This is because it is an environment that allows the execution of instructions written in JavaScript (JS), the same programming language used by any web browser to interpret the code written for clients. This means that by adopting Node.js, it was possible to create the entire platform by writing code in a single programming language. JS is also a relatively easy coding language to learn. In addition to Node, a series of open-source libraries useful for developing certain functionalities were used.

Firstly, the *Express.js* library was used to facilitate the scaffolding of the server, its connections, and the various middleware deemed necessary to structure client-server communications appropriately. In addition, the *node-postgres* library was used, which allows the server to interact with databases made in PostgreSQL, such as in the case of this project, facilitating the connection with them and writing SQL (Structured Query Language) queries directly within the program written in JavaScript.

In addition to the shared database, it was also necessary to structure adequate online file storage where images and documents saved from the platform could be stored and potentially host the relatively large files of BIM models in case of a lack of alternatives. For this type of functionality, Supabase, a web service, was chosen, which is also open-source and has few limitations in terms of database size, storage space, and the number of simultaneous connections.

**Figure 13:**
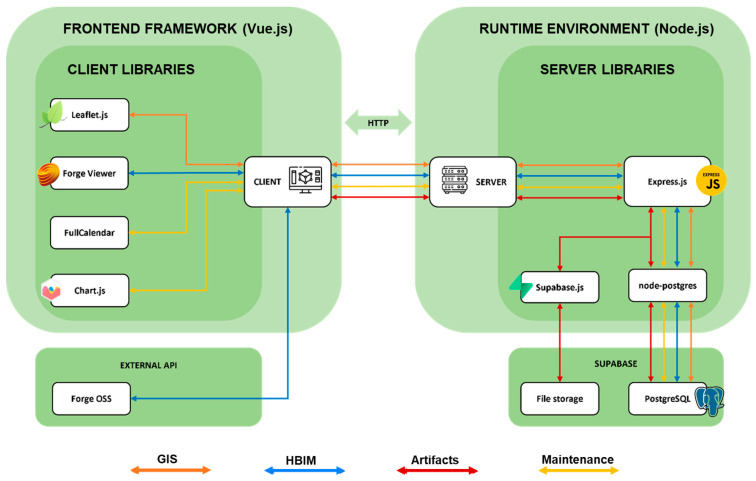
Structure of the data flow through the platform, libraries and services used.

Finally, as will be explained further for the client aspects related to visualization in the BIM environment, an Autodesk service called Forge (at the time of the project’s development: At the time the authors are writing, this service has been redirected to another one called APS, Autodesk Platform Services) was used to host the BIM models made in Revit within a system that allowed for their storage and conversion into formats compatible with libraries for visualization on the client. All of this is possible through a series of dedicated APIs (Application Programming Interfaces) made available by Forge, with which to interact with Autodesk servers and send requests and receive their respective responses (Figure 13).

#### 3.2.7. Frameworks, Libraries, and Structure of the Client Interface

From the client’s point of view (see Figure 13 above), since it is a web application, the languages used were basically three: HTML (HyperText Markup Language), CSS (Cascading Style Sheets), and JavaScript. In general, in web pages, HTML is used to define the structure of the page, CSS to define its graphic and style aspects, while the JavaScript language is used to establish the logic and behavior, therefore, the actual “program” [29].

In the case of relatively simple websites, it is sufficient to limit oneself to this division to produce each page. However, these are solutions that are not very scalable since as the complexity of the site grows, the difficulty of implementing new features grows exponentially. For this reason, for about ten years now, the use of “front-end frameworks” has become standard in web development workflows, which are JavaScript libraries developed for web application development that facilitate code management and the division of pages into “components”, containing both the structural and stylistic aspects, as well as the logic of the program, all assembled to compose the page in a single file [30].

Given the evident complexity of the designed platform, one of these frameworks, *Vue.js* (specifically version 3) [31], was chosen for the development of the client interface and its behavior. The choice was essentially motivated by the relatively better learning curve, as this framework has proven to be extremely developer-friendly, according to the authors’ judgment. In addition to Vue, numerous support libraries were used for the development of the front-end part of the platform. In particular, the *Leaflet.js* library was used to create a navigable map within the browser page, on which the geometries from the GIS part of the shared database can be positioned. For the BIM viewer, for which a three-dimensional virtual environment was necessary within the browser to render the models and allow exploration, the libraries of the aforementioned Autodesk Forge were used, which in turn rely on the *Three.js* library for the creation of the 3D environment on web pages. Finally, for the parts of the platform dedicated to operator users, in which it was necessary, as will be explained later, to include a calendar and specific graphs, the *FullCalendar* and *Chart.js* libraries were used.

At the interface structure level, the platform was divided into a series of thematic “tabs”: In particular, there are three Viewers, a Planner, and a Dashboard. Each tab has a main panel and a side panel called “Explorer”, containing additional functionality for interaction with the main panel.

#### 3.2.8. Communication Protocols between Client and Server

As mentioned, communications between client and server occur through requests with the HTTP protocol (HyperText Transfer Protocol). Without going excessively into the details of the protocol itself, basically, at certain user interactions with the interface (opening a certain tool, clicking on a certain button, etc.), given requests (i.e., GET: data request, POST: data sending or modification, DELETE: data deletion, etc.) are sent to the server at specific “endpoints” (i.e., baseUrl+/database/gis/levels/), based on which the Express middleware of the server has been programmed to execute certain queries or retrieve certain data from other external APIs (based on the same principle of requests on specific endpoints). When the server receives a specific request concerning data hosted within the database or interaction and/or modification with them, it performs a certain SQL query, prepared, and parameterized within the program, to be sent to the database.

The library that interacts with Express, responsible for sending the query to the PostgreSQL database and for subsequently receiving and transmitting the data back to the server, is *node-postgres*. It is, in fact, configured as a bridge between the server and the database, in the present case hosted on the Supabase service, through a connection that is established at the opening of the application. In the case of requests reserved only for authorized users, whether they are operators, managers, or administrators, a dedicated middleware has been set up on the Express server, which, before executing the requested operation, verifies the credentials of the user who performs it, through a check of technical cookies prepared at the time of access, which are sent together with the request itself.

#### 3.2.9. The Viewers

**Figure 14:**
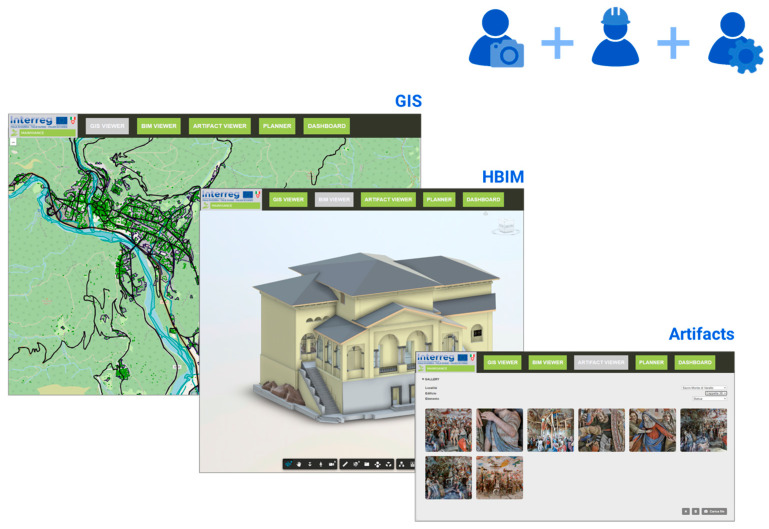
4MAIN10ANCE platform viewers.

The three viewers, in their simplified configuration, without any functionalities related to data insertion or modification, are accessible to any type of user, regardless of their role (Figure 14).

The first of these is the “GIS Viewer”, which is the GIS information viewer: It consists of a map on which there are some markers relating to the location of various significant locations (in the case of this project, the Sacri Monti of Northern Italy and Canton Ticino) and buildings belonging to these locations, for which there is a BIM model. By interacting with these markers, a request is sent to the server and to the Forge API to receive the model from the client and automatically display it on the three-dimensional viewer. In addition to these markers, the GIS Viewer also has a series of layers, each of which is linked to a specific GIS entity in the shared database: By interacting with these layers, it is possible to make visible on the map, following a specific request sent to the server when opening them, all the objects present on the database contained in certain selected entities. The objects can be points, lines, or polygons, and by interacting with each of them, it is possible to display their main information.

The second viewer, namely the “BIM Viewer”, is a 3D viewer for exploring and querying BIM models. Here it is possible to select, through a dropdown menu, the desired model and display it on the page, through a request to the platform, and subsequent response, towards the Forge API. In this viewer, users with a higher level of authorization, such as operators and managers, can access additional functionalities for registering master data, control, or intervention sheets related to one or more elements of the open BIM model. Such additional data are recorded in corresponding tables of the database and linked to the respective components of the models through a specific identifier, i.e., the id_main10ance exposed in the previous sections.

The last viewer is the one that has been called the “Artifact Viewer”, and it consists of an image gallery and a dedicated navigator in the side panel through which to filter the selection based on location, building, and category of objects belonging to LOD4. In this way, for example, by selecting among the filters the Sacro Monte of Varallo, the chapel 10 building, and the statue category, it is possible to display the gallery of all the statue images contained in that chapel. Such images are hosted inside the Supabase storage and received by the client through a request to the server with the necessary information. At the same time, each image also has a corresponding record within the table of its category, complete with a unique identifier and some general data. Moreover, in this case, authorized users such as operators and managers have access to additional functionalities for registering and managing data and the possibility of uploading new images.

#### 3.2.10. The Management Tools: Planner and Dashboard

The “Planner” is the first of the two tools accessible only to authorized users: The main panel contains a series of sections related to specific phases of the maintenance management process, while the Explorer displays a calendar on which scheduled activities are displayed. The sections in the main panel relate to the consecutive steps that the various assets of interest to the managing entity must go through according to the Maintenance Program. These have, therefore, been defined as *Planning*, *Programming*, *Execution*, and *History*. The first two sections, Planning and Programming, are only accessible to the managing user, while both can consult Execution and History.

The first section, *Planning*, allows the manager to draw up a rough plan for the maintenance of their assets, selecting by location, buildings, and categories: For each category, specific risk expressions (illustrated earlier) and related periodic check and routine maintenance activities to be carried out are linked. The manager establishes a frequency in months and a start date of the maintenance cycle for each risk expression. Once the planning of a certain group of assets is saved, a new record is created in the respective tables related to the different types of activities within the database: Within this record, a series of information related to them, an identifying reference of the applied risk expressions, as well as the list of unique IDs of the elements involved and a group ID are saved. In particular, the latter serves to allow the possible separation of some elements for targeted interventions and their subsequent reinsertion in the periodic cycle once realignment is complete within the same group from which they were extracted.

The conclusion of the planning phase also creates a specific card for each building and for each activity to be carried out in the *Programming* section. Here, the manager adds additional information on duration and estimated cost, the operator in charge of carrying out the activity, necessary materials and logistics, and any additional annotations. Here, the manager also has the option to modify the scheduled date for the activity, initially calculated automatically by the platform based on the periodicity established during planning. Once this additional information is saved, the corresponding record is updated in the database, and a new intervention card is created on the Execution section of the platform, visible to the assigned operator. In parallel, during these first two phases, an update is also made to the calendar in the Explorer: After planning is completed, the system automatically positions the events on the calendar based on the set frequency. Upon completion of the programming phase, these events are updated and definitively positioned on the confirmed dates.

In the *Execution* section, the operator can view the check or intervention cards for which they have been assigned, on which all detailed information entered up to this point is reported. The fact that each activity is linked to individual elements through the id_maintenance allows for a direct passage to the corresponding domain’s Viewer. This is achieved by the application’s decomposition of the id_maintenance into its parts: From the category information, the system goes back to the corresponding domain, and therefore, to the corresponding Viewer (LOD2: GIS Viewer, LOD3: BIM Viewer, LOD4: Artifact Viewer); the location and building components of the id allow the appropriate request to be sent to the server and receive the data (and any model requested from the Forge API) retrieved within the database; finally, the object identifier component allows for preselection of the elements of interest. In this way, the operator can consult the intervention card for which they have been assigned through the Execution section of the Planner and subsequently, through a specific functionality of the card, move to the Viewer of the elements of interest. From here, they can then compile the detailed activity sheet through the advanced functionalities of the Viewer’s Explorer.

Finally, after the operator has registered a completed activity, the platform updates the related data on the database, the activity card is transferred from the Execution section to the *History* section, where it becomes a real archive of interventions that grows over time, and the platform automatically creates a new activity to be scheduled, inserted in the Programming section and with pre-set timeframes based on the frequency given at the beginning of the maintenance cycle. The manager thus has the possibility to schedule such new future interventions. This so-called automatic scheduling, performed by the system based on registered interventions, is also based on a specific concept of algorithmic priority management, which will be discussed later.

The last tool provided by the platform is the so-called “Dashboard”, accessible only by the manager user, and which consists of a series of graphs, tables, and general summary indications on the consistency and status of the assets under their protection, on the interventions carried out, in progress and planned, and several other specificities that may be of interest to the manager in order to have an overview and make broad assessments on the progress of maintenance cycles and consider any variations. The final tool of the Dashboard thus constitutes a fundamental support for the management of the Planned Conservation Plan of the assets in charge of the manager. For this section of the platform, the client only sends requests related to the set of assets and receives the corresponding data from the server, which are rendered on the page through specific charts from the Chart.js library.

#### 3.2.11. The Priority Matrix of Interventions

**Figure 15:**
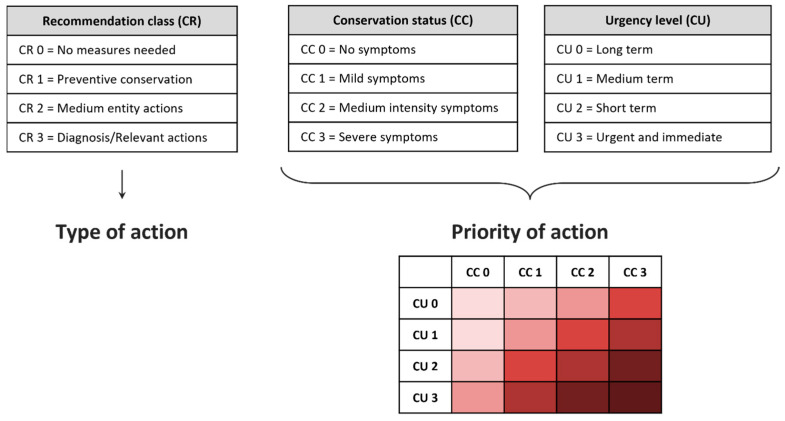
Schematic summary of the priority matrix for the automatic scheduling of conservation activities.

Among the data that the operator enters into the control activity forms, there are three fields of fundamental importance for managing the subsequent phases: “CC—Conservation status”, “CR—Recommendation class”, and “CU—Urgency level”, encoded within the Italian-European standard relating to the reporting of the conservation status of built cultural heritage [32]. Each of them presents four predetermined levels, to which a number in a pre-set range has been associated, in order of severity. The first of these parameters, CC, establishes which type of action will be automatically planned by a specific algorithm of the platform: A good conservation status corresponds to the scheduling of a new periodic regular activity, while in case of a non-good conservation status, a realignment activity is planned that can vary between corrective maintenance, extraordinary maintenance or restoration. Furthermore, it is also possible to record the CR and CU parameters in case of a non-good conservation status. In this way, the operator has the possibility to report the perceived cost of the intervention and with what urgency it should be carried out: The cross between these two parameters, through multiplication of their values, gives rise to a priority matrix (Figure 15), whose value is saved within the database along with the other data, and which allows establishing a priority order for the manager in the list of activities to be programmed on the Planner.

**Figure 16:**
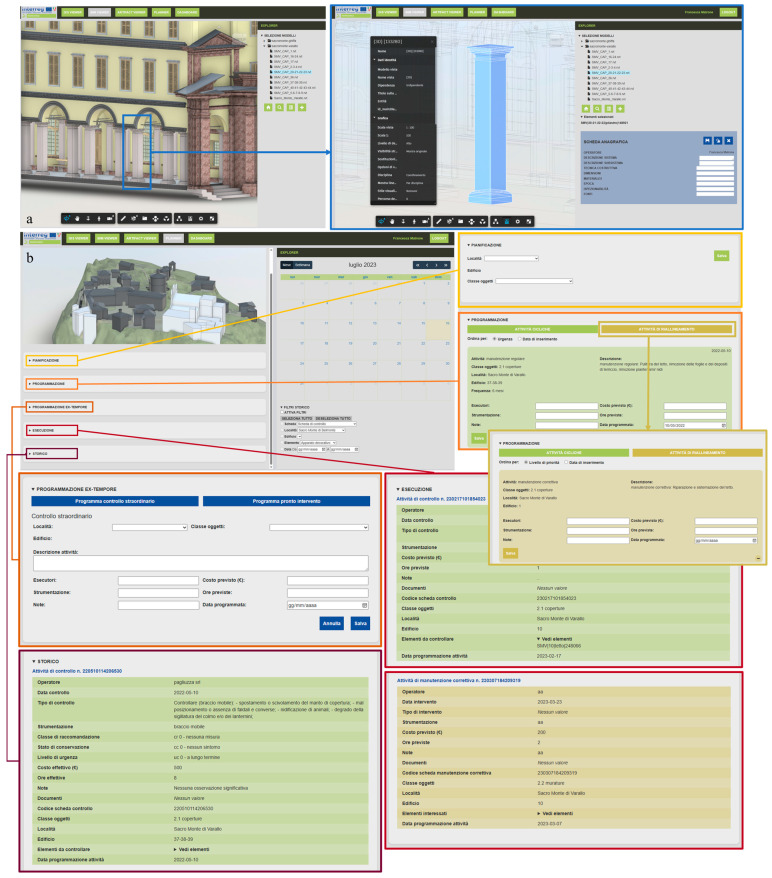
The BIM viewer (**a**) and the Planner (**b**) with the relative sections.

Figure 16 shows the possibility of inserting these parameters in the Planner.

First of all, it represents the chance to see the information on the individual architectural elements of the HBIM models and enter new ones thanks to the *Registry card* (Figure 16a). Then, in the Planner (Figure 16b), the sections of *Planning*, *Scheduling* (subdivided into cyclical and realignment activities as foreseen in the maintenance cycle), *Ex-tempore scheduling* (with the scheduling of extraordinary checks and emergency interventions), *Execution* (subdivided into control and corrective maintenance) and *History* of activities are visible. The above-mentioned parameters are visible in the form of this last section.

## 4. Discussion and Conclusions

In the present contribution, a novel HBIM-GIS tool for the management plans of the landscape, built, and movable heritage has been described. It is the main output of the Interreg MAIN10ANCE project, and it is freely available to the scientific community, as well as to professionals and management bodies.

The described results are unique because they allowed creating a common platform able to:

- incorporate the multifaceted needs and requests of heritage workers (restorers, artisans, maintainers, etc.) into a single interoperable database. In fact, the design process required numerous meetings between the partners and stakeholders to understand their necessities. The results of these meetings flowed into the 4 Main10ance platform and database structure;
- collect data at different scales (BIM and GIS);
- be visualizable through a 3D multi-scale representation of the cultural heritage (environment, architecture, and indoor objects), whose digitalization is based on an accurate 3D metric integrated survey;
- take into account the timing, history, and cost of the interventions, facilitating future planning activities;
- be easily queried and used by tourists and operators (with different levels of skills), thanks to a user-friendly interface accessible through tablet, smartphone, or PC.

In addition, it is open and mostly based on open software.

The set of technologies, languages, and protocols presented in this paper has allowed developing a tool that the authors hope can be implemented, integrated, and adopted by entities, administrations, and institutions, public or private, that need to manage, maintain, and safeguard cultural assets, and that intend to optimize the management of related conservation operations and activities. With this vision, any HBIM model created according to the project guidelines can be uploaded to the database via a web interface and used for the preservation of its respective physical asset according to the logic of the platform, thus also providing very useful feedback to the improvement of the platform itself. The 4MAIN10ANCE platform can be considered a prototype version, still to be tested and improved, but already equipped with multiple functionalities that make it more than a simple “proof of concept”. The heritage modeling phase remains one of the hot buttons of this methodology, as it is quite consuming in terms of effort, time, and resources. Prospects point the way toward artificial intelligence algorithms, thanks to which it will be possible to fully automate the modeling phases starting from a point cloud, segmented and classified through other specific algorithms. Imagining rapid technological advancement in the field of robotics and surveying tools as well, research must focus on the management of all these data according to the set goal.

The potential of the developed methodology and tool is manifold.

Since, to date, HBIM models are increasingly used and mandatory by international regulations in the case of contracts beyond a certain budget limit, this tool can integrate them and support their planned maintenance. Furthermore, it has been structured and programmed on the basis of the specific requests of restorers and the management body, therefore it is able to host a whole series of information necessary for them and not foreseen in other state-of-the-art platforms. In addition, it has been designed to be user-friendly and to be employed in situ even by non-expert operators.

Finally, being based on international standards, it can be easily adopted internationally and adapted to other case studies, being the database structure open and modifiable. This possibility is confirmed by the fact that it has already been chosen and used for the management of another widespread UNESCO asset: “Ivrea, industrial city of the 20th century”. To this end, some parts are being adapted.

On the other hand, it should be noted that the tool is, at the moment, in Italian, and this element can constitute a critical issue. However, in the case of third-party adoption, it would be easily translatable and adaptable due to its open structure.

Among the future developments of this work, there is the inclusion of further case studies to test its effectiveness and evaluate possible modifications on the basis of user requests.

In addition, there is the possibility of adding ways to visualize three-dimensional models through virtual reality tours so as to provide increasingly immersive experiences for tourists unable to reach and see the cultural heritage in person, but also the possibility of on-site visualization by maintenance workers, through augmented reality, of hard-to-find information by facilitating access to it through the superimposition of digital data on top of reality.

Another important aspect for the future development of the research consists of the interoperability related to software and standards. Indeed, this research started some years ago, then, version 2 of the CityGML standard was adopted. In the last year, a new version has been launched. Its potentiality consists in the harmonization with the INSPIRE standard building elements, in the possibility to include point clouds from the 3D metric survey (photogrammetric or Lidar ones), and in the integration in the level of detail 3 and 4 of BIM models. Hence, possible future development of the platform could include this new standard version revising the structure of the database but maintaining existing and stored maintenance data. The other interoperability technical aspect regards the visualization of BIM and GIS models in a unique viewer, with different zooming levels. At the moment, only a few commercial solutions, such as ESRI ArcGIS Pro, provide the possibility to insert and visualize BIM data into the GIS platform, allowing the 2D and 3D visualization in the same viewer. One of the innovative aspects of the present research regards the connection of BIM models with GIS features. The limit of the present study and its demonstrator is related to the fact that in the demonstrator, there are two connected platforms for visualizing GIS and BIM 2D and 3D data. Future improvements to the platform could consider a unique BIM-GIS viewer.

## Data Availability

https://github.com/EmmanueleIacono/MAIN10ANCE_Viewer_DEMO (accessed on 23 June 2023).
